# Major basic protein and eosinophil peroxidase support microfilariae motility inhibition by eosinophil ETosis

**DOI:** 10.1371/journal.pntd.0012889

**Published:** 2025-03-03

**Authors:** Pia Philippa Schumacher, Jesuthas Ajendra, Benjamin Lenz, Frederic Risch, Alexandra Ehrens, Celia Nieto-Pérez, Marianne Koschel, Tilman Aden, Achim Hoerauf, Marc P. Hübner

**Affiliations:** 1 Institute for Medical Microbiology, Immunology and Parasitology, University Hospital Bonn, Bonn, Germany; 2 German Center for Infection Research (DZIF), Partner Site Bonn-Cologne, Bonn, Germany; National Institutes of Allergy and Infectious Diseases, NIH, UNITED STATES OF AMERICA

## Abstract

Eosinophils are a hallmark of filarial infections. They are primary effector cells and can attack filariae by releasing extracellular traps that contain toxic cationic proteins, such as eosinophil peroxidase and major basic protein. Previous studies demonstrated that the extracellular traps of eosinophils are induced by the microfilariae of *Litomosoides sigmodontis* and that they inhibit their motility. In this project, we aimed to investigate the role of these cationic proteins during the extracellular trap-mediated immobilization of microfilariae. Our results indicate that extracellular DNA traps from knockout mice that lack eosinophil peroxidase or major basic protein are significantly less able to immobilize and kill microfilariae. Accordingly, the addition of these cationic proteins to *in vitro* cultures inhibited microfilariae motility in a dose-dependent manner. Moreover, we examined eosinophils from the natural host, the cotton rat *Sigmodon hispidus*. While eosinophils of cotton rats release DNA after stimulation with PMA and zymosan, microfilariae did not trigger this effector function. Our work shows that eosinophil granule proteins impair the motility of microfilariae and indicate significant differences in the effector functions of eosinophils between the mouse model and the natural host. We hypothesize that the absence of DNA nets released by cotton rat eosinophils in response to microfilariae may explain the higher microfilarial load and longer patency of the natural host.

## Author summary

Filarial nematodes are thread-like worms that can cause debilitating diseases such as river blindness (onchocerciasis) or elephantiasis (lymphatic filariasis). Similar to most helminth infections, filarial infections lead to eosinophilia, an increase of eosinophil granulocyte numbers in the peripheral blood. Although eosinophils have been shown to contribute actively to the protective immune responses, the underlying mechanisms are not yet fully understood. In the present study, we investigated the protective effect of the eosinophil granule proteins major basic protein (MBP) und eosinophil peroxidase (EPO) against the filarial progeny, the microfilariae, of the rodent filarial nematode *Litomosoides sigmodontis*. Eosinophils lacking MBP or EPO inhibited microfilariae motility *in vitro* to a lesser extent than wild-type eosinophils. Consistent with these results, the addition of MBP and EPO inhibited microfilariae motility *in vitro*. Additionally, we demonstrate that eosinophils from the natural host of *L. sigmodontis*, the cotton rat, did not inhibit microfilariae motility in the same way, indicating either intrinsic differences among cotton rat and mouse eosinophils or an adaptation of the parasite to its natural host.

## Introduction

Eosinophils are a hallmark of helminth infections, exhibiting both protective and pathological functions in the immune response to filarial infections [1–3]. Eosinophils produce crystalloid granules that contain major basic protein (MBP), eosinophilic cationic protein (ECP), eosinophil-derived neurotoxin (EDN) and eosinophil peroxidase (EPO), which are associated with the killing of the filarial progeny (microfilariae (MF)) and drug-induced adverse events in filariasis patients.

During onchocerciasis (aka river blindness), the death of *Onchocerca volvulus* MF in the skin and the cornea can lead to dermatitis and vision loss, respectively. While the results on the role of eosinophils and their granules in the development of keratitis are controversial [4–6], their impact on dermatitis is more clear. Increased inflammatory immune responses against the MF, as observed in hyper-responsive onchocerciasis patients or following DEC treatment, are associated with eosinophil responses and significantly elevated ECP, EDN and MBP levels [7–9]. Such an increased eosinophil recruitment and activation was also observed in humans and primate animal models following treatment-induced killing of MF of the African eye worm *Loa loa* [10,11]. Similarly, temporary residents, who more frequently develop clinical symptoms during infections with *L. loa* and following DEC treatment are associated with higher eosinophil counts and eosinophil granule protein levels in comparison to endemic individuals [12,13]. Despite these findings, treatment with anti-IL-5 antibodies to reduce eosinophil responses following DEC treatment in loiasis patients did not reduce the adverse events [14]. Thus, more work is required to decipher the role of eosinophils and their granule proteins during filarial pathology. In the case of tropical pulmonary eosinophilia (TPE), a severe disease that affects some patients with lymphatic filariasis, eosinophil responses to MF are the cause of severe lung inflammation associated with increased levels of EDN and MBP [15–18].

With regard to protective immune responses to filariae, studies on diverse filarial species and animal models have demonstrated that eosinophils support the clearance of adult worms [1–3,19,20]. Interestingly, in nodules containing adult *Onchocerca* filariae, it was shown in cattle as well as humans that neutrophils primarily surround viable adult worms and release extracellular DNA traps. Only after antibiotic clearance of the *Wolbachia* endosymbionts of filariae did eosinophils infiltrate the nodules and degranulate, supporting a role in worm killing [21–23]. Eosinophils also contribute to the clearance of MF, as was shown by injection of *Brugia malayi* MF in naïve wildtype (WT) and eosinophil-deficient PHIL mice [24]. Of note, lack of MBP and EPO did not impact the survival of *B. malayi* MF after injection in naïve mice in comparison to WT controls [24]. This is in contrast to *in vitro* findings, where MBP, ECP, EDN, and EPO were shown to kill *B. malayi* and *Brugia pahangi* MF [25]. Furthermore, data from a mouse model with experimentally injected *Onchocerca lienalis* MF indicated that mice develop resistance to a re-injection with MF, which was dependent on the presence of eosinophils [26].

In line with the results described above, *Litomosoides sigmodontis* infections in eosinophil-deficient dblGATA mice and IL-5 deficient mice resulted in a higher worm burden, prolonged worm survival and increased MF load [27,28]. In line, eosinophilic IL-5 transgenic mice had a significantly reduced *L. sigmodontis* adult worm burden [29] suggesting an essential role of eosinophils in host protection. The cytotoxic granule proteins MBP and EPO have been linked to susceptibility to infection with *L. sigmodontis* before [30], as mice on the “resistant” 129/SvJ background deficient for either EPO or MBP developed a significantly higher worm burden than 129/SvJ WT mice [30]. More recently, our group demonstrated that eosinophils release extracellular DNA traps (EETosis) during filarial infections [31,32]. *In vitro,* eosinophils undergo ETosis upon MF stimulation, which inhibits MF motility in a contact-dependent manner. EETosis has also been shown to facilitate MF clearance *in vivo* [31].

Next to citrullinated histones, eosinophil extracellular traps contain the granule proteins ECP, EDN, MBP, and EPO [1]. In this study, we investigated whether the co-localized eosinophil granule proteins MBP and EPO are required for the EETosis-mediated immobilization and potential killing of MF. By utilizing eosinophils of MBP- and EPO-deficient mice and co-cultures with MBP and EPO, we found that these granule proteins indeed contribute to eosinophil-mediated MF immobilization and may play a role in MF killing. Interestingly, studies with the natural cotton rat host *Sigmodon hispidus* revealed that their eosinophils are less able to immobilize MF, suggesting a better adaptation of *L. sigmodontis* to its natural host compared to laboratory mouse strains or intrinsic differences in eosinophil effector functions of both host species. In conclusion, our study provides a deeper insight into the effector functions of eosinophil and the phenomenon of EETosis during filarial infections.

## Methods

### Ethics statement

All experiments were performed according to EU directive 2010/63/EU and approved by the appropriate authority Landesamt für Natur-, Umwelt- und Verbraucherschutz (LANUV, Recklinghausen, Germany; AZ 81-02.04.2020.A103; 81-02.04.40.2022.VG029).

### Mice/ Cotton rats

129/SvJ wildtype (WT) mice were purchased from Charles River Laboratories. EPO and MBP-knock-out (KO) mice were originally obtained from Nancy A. Lee and James J. Lee, Department of Biochemistry and Molecular Biology, Mayo Clinic Scottsdale, Scottsdale, Arizona, USA, and were bred at the “Haus für Experimentelle Therapie” of the University Hospital Bonn, Germany. Mice of both sexes were used.

Cotton rats (*Sigmodon hispidus*) were bred at the Institute for Medical Microbiology, Immunology and Parasitology. Female cotton rats were used. Mice and cotton rats were housed in individually ventilated cages with unlimited access to food and water and a 12-h day/night cycle. Knockout of MBP and EPO was confirmed by PCR.

(EPO-KO: P1: 5′-TGAAACCCCCAAACTGACGG-3′, P2: 5′-ACAGAGCTAAGCGGGACGTG-3′, P3: 5′-CATCGAGCGAGCACGTACTC-3′; MBP-KO: P1: 5′-ATGCCACTGTGAGACAGGGTAAGG-3′, P2: 5′-CAGATGAAGAGCAGACGCTC-3´, P3: 5′-GAACCAGCTGGGGCTCGAG-3′)

### Microfilariae isolation

MF were isolated as described before [33]. In brief, cotton rats infected with *L. sigmodontis* were anaesthetized with isoflurane and blood was collected. The collected blood was then diluted 1:2 with Advanced RPMI 1640 medium (Gibco, ThermoFisher Scientific, California, USA) containing 10% FBS (Gibco, ThermoFisher Scientific, California, USA), 1% L-glutamine (Gibco, ThermoFisher Scientific, California, USA) and 1% penicillin/streptomycin (Gibco, ThermoFisher Scientific, California, USA). A sucrose-percoll gradient was used for MF purification as previously described [33]. Therefore, a gradient of 30% and 25% percoll-sucrose-solution (Cytiva, Global Life Sciences Solutions USA, Marlborough USA) was added to a 15 mL tube and the diluted blood was layered on top. The gradient was centrifuged without breaks at 340 g for 30 min at room temperature and MF were collected from the layer between the 25 and 30% solution. The purified MF were washed 3 times with Advanced RPMI medium and then diluted in 1 mL medium and quantified with a Neubauer counting chamber.

### Eosinophil generation

Bone marrow from naïve SvJ/129, EPO-KO and MBP-KO mice and naïve cotton rats was used to generate bone marrow-derived eosinophils as previously described [31,32]. After red blood cell lysis (Invitrogen, eBiosience ThermoFisher Scientific, California, USA) for 5 min with 1mL of RBC lysis buffer diluted 1/10 in dH_2_O, bone marrow cells were counted using the CASY TT- cell counter system. 1 x 10^6^ cells/mL were seeded in pre-warmed Advanced RPMI medium supplemented with 20% FBS, 1% penicillin/streptomycin (Gibco, ThermoFisher Scientific, California, USA), 0.1% gentamycin (Gibco, ThermoFisher Scientific, California, USA), 2.5% HEPES (Gibco, ThermoFisher Scientific, California, USA) and 1% Glutamax (Thermo Fisher Scientific GmbH, Germany). The cells were cultured for the first 4 days with stem cell factor (SCF, 100 ng/mL) (Peprotech, Rocky Hill, USA) and FMS-like tyrosine kinase 3 ligand (FLT3L, 100 ng/mL) (Peprotech, Rocky Hill, USA). The growth factors were then exchanged with IL-5 (20 ng/mL) (Peprotech, Rocky Hill, USA). Half of the medium was replaced every other day. The cell culture flask was replaced on day 6 for cotton rat and day 8 for mouse eosinophils. Cells were harvested after 12 days for mouse eosinophils and 8-10 days for cotton rat eosinophils.

The cells were then checked for purity by flow cytometry for mouse eosinophils with the surface marker SiglecF (APC-Cy 7 rat anti-mouse Siglec-F, Dye: APC-Cy7, Clone: E50-2440, BD Biosciences, Franklin Lakes, USA) or Diff Quick (RAL Diff-Quick kit, RAL DIAGNOSTICS, Martillac, France) staining for cotton rat eosinophils as there are no cross-reactive antibodies for cotton rats, which could be used for flow cytometry. The resulting purities of the cell cultures were 91.0 - 97.3% for WT SvJ eosinophils, 90.3 - 92.5% for MBP-deficient eosinophils, 90.7 – 97.6% for EPO-deficient eosinophils and 96.4 - 97.7% for the cotton rat eosinophils.

A separate experiment was performed to identify additional cell populations that were present alongside the bone marrow-derived eosinophils. The following antibodies were used (antibodies were sourced from Biolegend, San Diego, USA unless stated otherwise): αCD11b-Al700 (clone: M1/70), αLy6G-PE-Cy7 (clone: 1A8), αF4/80-PerCP-Cy5.5 (clone: BM8), αCD4-Al488 (clone: RM4-5), αCD19-APC (clone: 6D5), αCD8-APC-Cy7 (clone: 53-6.7), αCD1-BV510 (clone: 145-2C11), αCD11c-BV605 (clone: N418). αCD3-BV510 (clone: 145-2C11), αI-A/I-E-BV421 (clone: M5/114.15.2) and αSiglecF-PE (E50-2440, obtained from BD Biosciences, Franklin Lakes, USA). In this experiment, eosinophils constituted 95.9% of cells, neutrophils constituted 1.46% of cells and the remaining cell population frequencies ranged between 0.02% and 0.56% and were made up of DCs and monocytes (S1 Fig). Antibodies to differentiate mature from immature granulocytes were not included in the analysis. Diff Quick staining of cotton rat eosinophils revealed a proportion of ~ 2% non-eosinophils. These non-eosinophil cells represented neutrophil granulocytes, lymphocytes, plasma cells and other cell populations (S2A Fig).

### Establishment of co-culture

Co-cultures were prepared in F-bottom 96-well plates with 1 x 10^5^ eosinophils as well as 2.5 x 10^3^ MF or 1 x 10^4^ MF per well in Advanced RPMI medium. The medium was supplemented with 10% FBS, 1% penicillin/streptomycin (Gibco, ThermoFisher Scientific, California, USA), 0.1% gentamycin (Gibco, ThermoFisher Scientific, California, USA), 2.5% HEPES (Gibco, ThermoFisher Scientific, California, USA) and 1% Glutamax (Thermo Fisher Scientific GmbH, Germany). As positive control, eosinophils were stimulated with phorbol-12-myristat-13-acetat (PMA, 0.5 µg/mL) (Cayman Chemical, Michigan, USA) and zymosan (0.5 mg/mL) (InvivoGen, Toulouse, France). In a subset of experiments, eosinophils were co-cultured with 0.1, 1 or 10 ng/mL MBP or EPO (EPO: LS Bio, Lynwood Australia, MBP: My Bio Source, San Diego, USA). The co-cultures were incubated at 37°C for either 24 hours (DNA release) or 72 hours (motility assays). After 72 hours, eosinophil viability dropped to 86-90%, preventing further analysis. As an internal control, a subset of samples were treated with DNase (20 U/mL) to ensure that the measured release was indeed DNA.

### Motility assay

For the motility assay, MF were scored after 4, 24, 48, and 72 hours. The motility of 20 MF per well were scored from three separate wells using a bright-field microscope (Leica Model DMIL LED, Leica Microsystems CMS GmbH, Wetzlar, Germany, magnification 10x and 20x). The motility score ranged from 0 to 4 with 0 meaning no movement and 4 representing continuous and fast motility. MF with a score of 3 showed slower but continuous movements, while a score of 2 indicated slow and discontinuous movements. MF with a score of 1 only showed sporadic movements at the ends. The assessment was done in a blinded manner.

### DNA quantification

For DNA quantification, the DNA Quant-iT kit (Invitrogen by Thermo Fisher Scientific GmbH, Germany) was used as previously described [31,32]. Therefore, the samples were treated with micrococcal nuclease for 15 min at 37°C to break up the released DNA networks for better measurement. This process was stopped with 1 mM EDTA. After centrifugation at 400 g for 10 minutes at 4°C, 20 μl of sample and 10 μl of the supplied standard were added to a 96-well F-bottom plate and 100 μl of the 1/200 diluted DNA Quant-iT-dsDNA HS reagent was added. The absorbance was measured using a Tecan Infinite 200 Pro plate reader (Tecan Trading AG, Switzerland) at excitation/emission wavelengths of 485/535 nm. DNA was quantified via the absorbance values of the standard.

### Fluorescence microscopy

1 x 10^5^ murine bone marrow-derived eosinophils were co-cultured in Advanced RPMI 1640 medium containing 1% penicillin, 1% streptomycin, 0.1% gentamycin, 1% L-glutamine, 10% FBS and 20 ng/mL of murine recombinant IL-5 with 1 x 10^4^ MF in 96 well plates for 24 and 72 h. After incubation, plates were centrifuged for 10 minutes with 400 g at RT and the supernatant was removed. 200 µl PBS including 0.002% propidium iodide was added to each well and incubated for 20 min at 37°C with 5% CO₂. The plates were then centrifuged at 400 g for 6 min at room temperature and 50 µl were replaced with fresh PBS. For DAPI staining, 15 mm coverslips were coated in a 24-well plate with 400 µl poly-L-lysine/well overnight at 4°C. The coverslips were then washed three times and the supernatant was discarded. The slides were then sterilized for 20 min under UV light and air-dried. For each well, 1 x 10^5^ bone marrow-derived eosinophils of the cotton rat were added and incubated with PMA (Cayman Chemical, Michigan, USA), zymosan (InvivoGen, Toulouse, France) or MF for 24 hours at 37°C. Advanced RPMI medium supplemented with 2.5% FBS, 1% penicillin/streptomycin (Gibco, ThermoFisher Scientific, California, USA), 0.1% gentamycin (Gibco, ThermoFisher Scientific, California, USA), 2.5% HEPES (Gibco, ThermoFisher Scientific, California, USA) and 1% Glutamax (Thermo Fisher Scientific GmbH, Germany) was used. Afterwards the cells were fixed with 4% paraformaldehyde (ThermoFisher Scientific, California, USA) and washed with H_2_O. Eosinophils were then permeabilized with 2% Bovine Serum Albumin (PAN Biotech, Passau, Germany) and 0.1% Triton-X (ThermoFisher Scientific, California, USA) overnight at 4°C. After washing with PBS, the cells were stained with 1/1000 diluted DAPI (300 nm, ThermoFisher Scientific, California, USA) with or without 5 µg/ml wheat-germ-agglutinin in PBS (ThermoFisher Scientific, California, USA) for 20 min at room temperature in the dark. The reaction was stopped through the addition of H_2_O and the supernatant was discarded. The cells were analyzed using the Axio Observer 7 with the Zen2.6 software (Carl Zeiss Microscopy Deutschland GmbH, Germany).

### Statistics

Prism 7.0 (version 7.0c, GraphPad Software) was used for statistical analysis. The data were tested for normality using the Shapiro-Wilks test. Differences between experimental groups for non-parametric data were determined using the Kruskal-Wallis test, followed by Dunn’s multiple comparisons test. The differences between the experimental groups for parametric data were assessed by one-way-ANOVA followed by Sidak’s post-test or two-way-ANOVA followed by Bonferroni post-test. The data are presented as scatter dot plot with mean and SEM.

## Results

### Inhibition of microfilariae motility by eosinophils is impaired in the absence of MBP and EPO

To investigate the contribution of MBP and EPO in immobilizing MF, bone marrow-derived eosinophils of MBP-KO, EPO-KO, and 129/SvJ WT control mice were co-cultured with isolated MF from cotton rats for 72 hours and MF motility was monitored over time.

**Fig 1:**
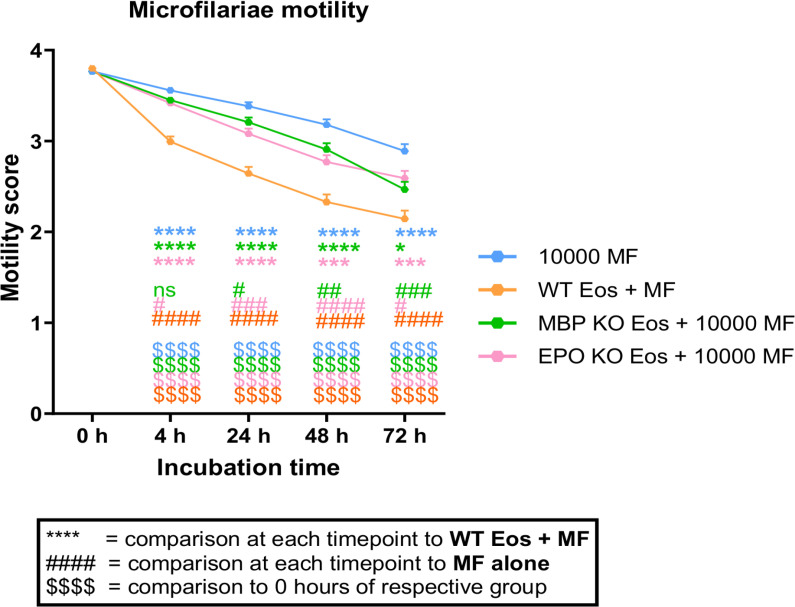
MBP and EPO contribute to inhibition of MF motility. Motility of 10000 MF that were co-cultured with bone marrow-derived eosinophils from MBP-KO, EPO-KO, and SvJ wildype (WT) control mice for up to 72 h. Pooled data from 5 independent experiments with a total of n = 300 microfilariae (MF) (3 wells per experiment with 20 MF each). Motility was documented per condition, not per well. Data were analyzed using two-way ANOVA followed by Bonferroni multiple comparisons test (A-B). *,# p<0.05, ## p<0.01, ***,### p<0.001, ****,$$$$,#### p<0.0001, ns = not significant.

Already after 4 hours of culture, MF cultured with eosinophils from WT mice showed significantly decreased motility (mean score of 2.99, continuous slow movement) compared to MF cultured with eosinophils from mice lacking MBP or EPO, or MF alone. MF from the latter groups still showed a continuous fast movement (EPO-KO mean score of 3.42; MBP-KO mean score of 3.45; MF alone mean score of 3.56; Fig 1).

At later time points (24 h, 48 h, and 72 h), MF motility decreased under all conditions compared to MF-only controls. However, WT eosinophils reduced MF motility significantly more than eosinophils from MBP- and EPO-KO mice (Fig 1). In line, completely immobile MF as well as MF staining positive for propidium iodide were more frequent in co-cultures with WT eosinophils compared to MBP-KO and EPO-KO eosinophil co-cultures, indicating a potential impact of MBP and EPO on MF killing (S3, S4A-C and S5A-B Figs). Taken together, this data demonstrates that both MBP and EPO are important for the eosinophil-dependent immobilization of MF.

### DNA release in mice lacking EPO or MBP is similar to WT controls

**Fig 2:**
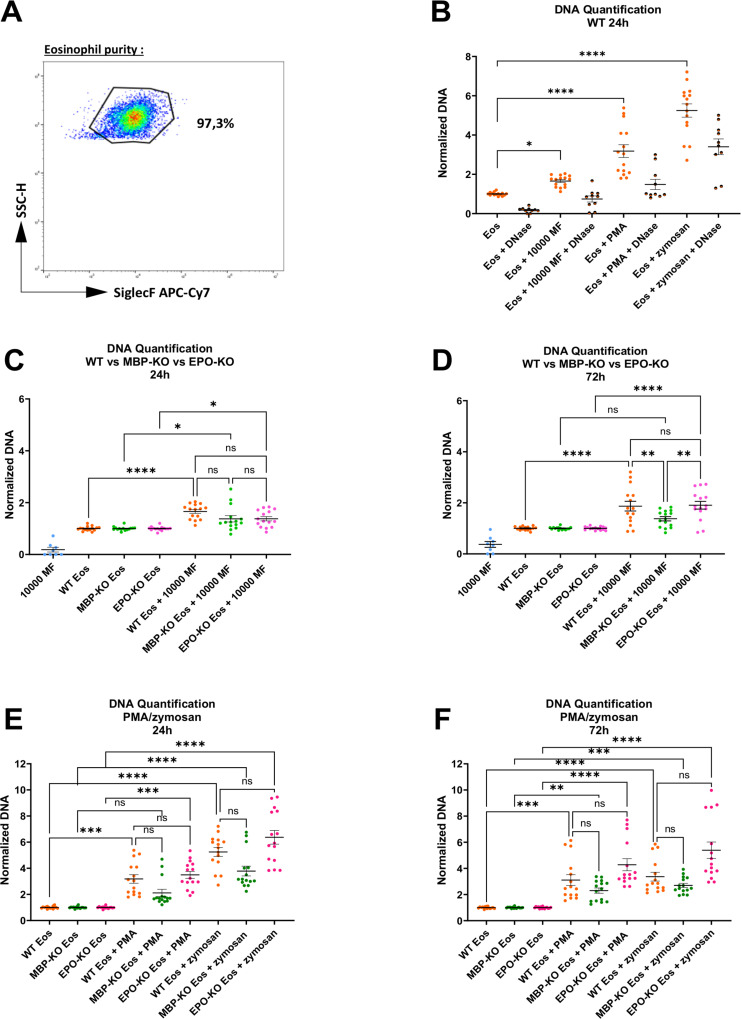
Similar DNA release of eosinophils of mice lacking EPO or MBP and WT controls. Representative flow cytometry-plot displaying the purity of bone marrow-derived eosinophils. Eosinophils were identified as SiglecF+ and SSC-H high (A). Normalized DNA in supernatant of bone marrow-derived eosinophils from SvJ wildtype (WT) mice stimulated for 24 h with 10000 microfilariae (MF), PMA, or zymosan (B). Pooled data from 5 independent experiments with a total of n = 300 MF (3 wells per experiment with 20 MF each). Normalized DNA in supernatant of bone marrow-derived eosinophils from SvJ WT, MBP-KO and EPO-KO mice stimulated for 24 h (C,E) and 72 h (D,F) with 10000 MF or PMA and zymosan. Pooled data from 5 independent experiments with a total of n = 300 MF/ 15 wells. Data were analyzed using Kruskal-Wallis test followed by Dunn´s multiple comparisons test for selected groups for non-parametric data (B-F). Data is shown as median with interquartile range (B) and Min to Max (C-F). *p<0.05, **p<0.01, ***p<0.001, ****p<0.0001, ns = non-significant.

To confirm that the reduced ability of eosinophils from mice lacking EPO or MBP to inhibit MF motility was not due to an impaired ability to undergo EETosis, we next examined the presence of free extracellular DNA in the culture supernatants from the experiments shown in Fig 1. Flow cytometric analysis of the eosinophils revealed a purity of >90%, which was comparable for all mouse strains (Figs 2A and S1). The eosinophils were cultured for 24 h with MF and the known EETosis inducers zymosan and PMA as positive controls. As shown in Fig 2B, MF lead to DNA release, while stimulation with PMA and zymosan induced the highest DNA release in this setup. As expected, DNase treatment reduced the DNA amount (Fig 2B). When comparing extracellular DNA release by eosinophils derived from EPO-KO and MBP-KO mice with 129/SvJ WT control eosinophils, similar though not identical patterns for MF-induced DNA release were observed for eosinophils from all three-mouse strains after 24 h (Fig 2C). After 72h, DNA was also released after stimulation with MF from eosinophils of all three mouse strains (Fig 2D). Notably, MBP-KO eosinophils released significantly lower amounts of DNA compared to WT and EPO-KO eosinophils after stimulation with MF (Fig 2D). No statistical significant difference in DNA release in response to stimuli like PMA and zymosan was observed among the three groups after 24 or 72 hours (Fig 2E-F). In general, MBP-deficient eosinophils showed the lowest DNA release at both time points, although this difference did not reach statistical significance. ELISAs to detect EPO and MBP were performed from cell culture supernatants from eosinophils stimulated with PMA, zymosan and MF, but the levels were below the detection limit.

In summary, these results show that stimulation with MF leads to DNA release in both WT as well as EPO and MBP KO mice, although MBP KO eosinophils showed slightly lower DNA release in some conditions. One possible explanation for this observation is the sheer amount of MBP that is present in eosinophils under normal circumstances. MBP usually makes up about half of all proteins in eosinophils and, as a result, eosinophils from MBP KO mice might require less DNA to transport their granule proteins out of the cell during EETosis. Nevertheless, both granule proteins contributed to the reduction of MF motility, confirming our hypothesis that both proteins contribute to MF immobilization in mice.

### MBP and EPO inhibit the motility of microfilariae *in vitro* in a concentration dependent manner

The results of the previous experiments indicated that the DNA inside the DNA traps is not solely responsible for the immobilization of MF. Instead, it seems likely that a significant function of the released DNA is dedicated to the guided transportation of granule proteins directly to the immobilized pathogen. To investigate the direct influence of MBP and EPO on MF motility, co-cultures with different concentrations of both proteins were performed in the absence or presence of WT and KO eosinophils.

**Fig 3:**
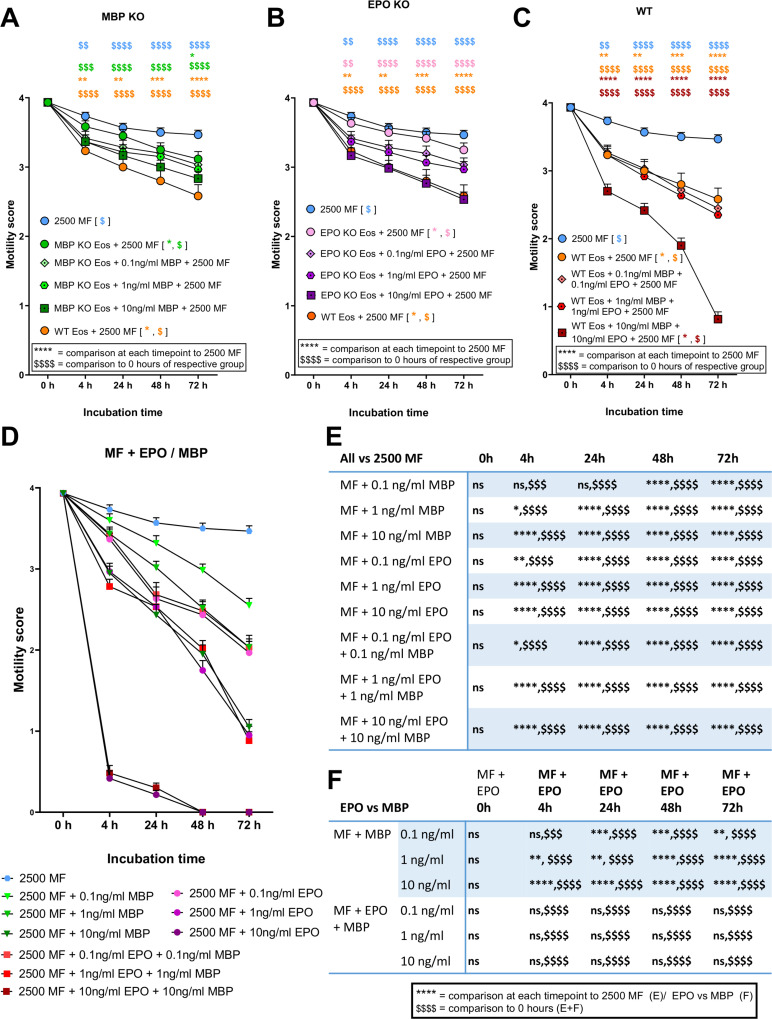
MBP and EPO inhibit microfilariae motility in a concentration-dependent manner *in vitro.* Motility of microfilariae (MF) that were co-cultured with bone marrow-derived eosinophils from MBP-KO (A), EPO-KO (B), and SvJ wildtype (WT) controls (C) and treated with recombinant MBP and EPO at indicated concentration for 4 h, 24 h, 48 h, and 72 h with 2500 MF (D). Statistical results of the data shown in Fig 3 D (E-F). Data from a single experiment with a total of n = 60 MF/ 3 wells. Data were analyzed using two-way ANOVA followed by Bonferroni multiple comparisons test. (A-D). * p<0.05, **,$$ p<0.01, ***,$$$ p<0.001, ****,$$$$ p<0.0001, ns = non-significant.

Both MBP (Fig 3A) and EPO (Fig 3B) enhanced the MF motility inhibition in the presence of the respective MBP- and EPO-KO eosinophils in a dose-dependent manner. MBP- and EPO-KO eosinophils achieved a similar MF motility inhibition with 10 ng/ml MBP and EPO (slow continuous to discontinuous MF movements, mean score of 2.83 and 2.53, respectively) as the co-culture with WT eosinophils (mean score of 2.58) after 72h. The addition of MBP and EPO to WT eosinophils further reduced the MF motility in a dose dependent manner, reaching a MF motility mean score of 0.82 after 72 hours of co-culture with 10 ng/ml of both proteins, indicating sporadic movement at the distal end of the MF (Fig. 3C). Finally, we examined whether MBP and EPO inhibit MF motility in the absence of eosinophils. Indeed, MBP and EPO inhibited MF more efficiently in the absence of eosinophils, resulting in complete inhibition of MF motility after 72 h of co-culture with 10 ng/ml EPO plus 10 ng/mL MBP or 10 ng/ml EPO alone (Fig 3D-F). The addition of 10 ng/ml MBP led to sporadic, distal movements of MF (mean score 1.05). The comparison of MF motility inhibition by MBP and EPO indicate that EPO inhibited MF motility significantly more than MBP (Fig. 3F). These results show that EPO and, to a lesser extent, MBP inhibit the motility of MF *in vitro* by themselves even in the absence of eosinophils.

### Eosinophils of the natural host are impaired in their response to microfilariae

**Fig 4:**
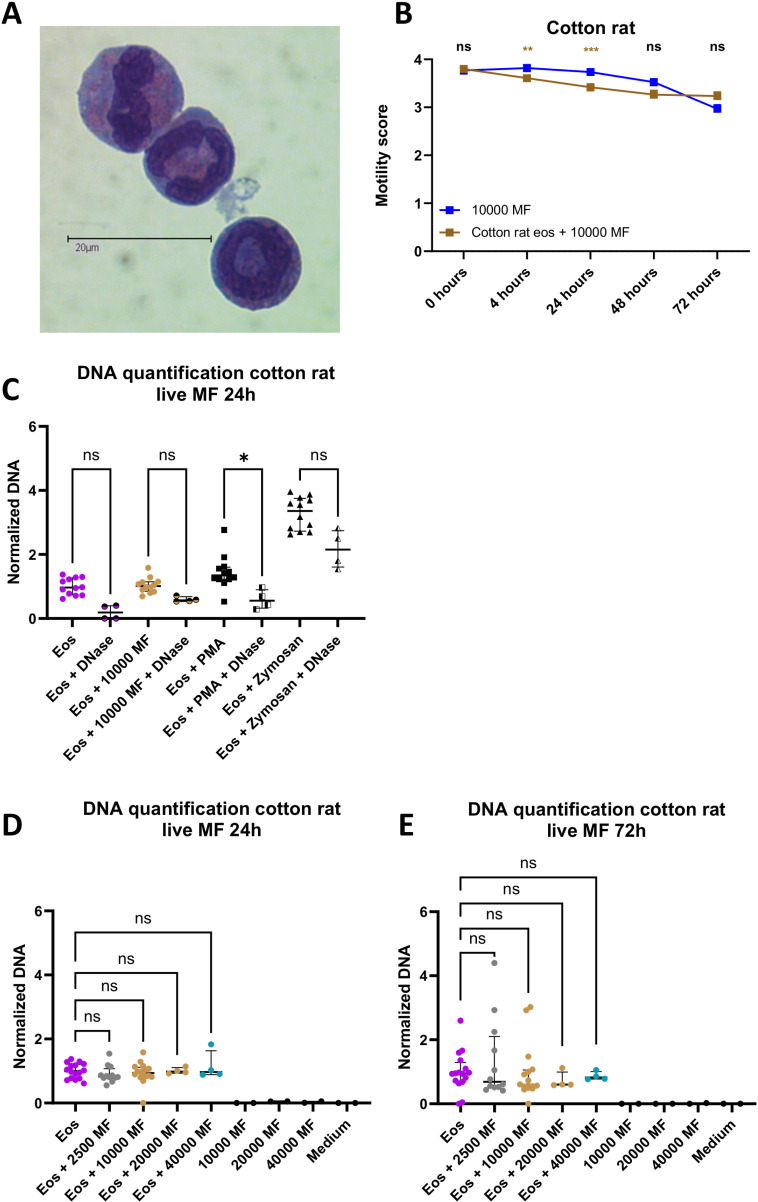
Eosinophils of the natural host do not release DNA traps in response to microfilariae and do not reduce microfilariae motility. Quick Diff staining of bone marrow-derived eosinophils from a cotton rat (A). Motility of 10,000 microfilariae (MF) co-cultured with bone marrow-derived cotton rat eosinophils (Eos) at 4 h, 24 h, 48 h, and 72 h (B). Free DNA in supernatant of bone marrow-derived cotton rat eosinophils stimulated for 24 h with 10,000 MF, PMA, or zymosan (C). Representative data from 2 experiments. Free DNA in supernatant of bone marrow-derived cotton rat eosinophils stimulated for 24 h (D) or 72 h (E) with 2500, 10,000, 20,000 or 40,000 MF, eosinophils or both. Pooled data from 1-3 independent experiments with a total of n= 80-320 MF/ 4-16 wells (D-E). Data were analyzed using two-way ANOVA followed by Bonferroni multiple comparisons test (B). Data were analyzed using Kruskal-Wallis test followed by Dunn´s multiple comparisons test for selected groups for non-parametric data (C-E). Data is shown as median with interquartile range (C-E). *p<0.05, **p<0.01, ***p<0.001, ****p<0.0001, ns = non-significant.

The natural host of *L. sigmodontis* is the cotton rat (*Sigmodon hispidus*). In contrast to the BALB/c mouse strain, in which the infection lasts up to 100 days and approximately 50% of the animals develop microfilaremia (S6A Fig) [34–36], almost all cotton rats develop microfilaremia with high MF levels and remain infected for more than one year (S6B Fig) [37]. To investigate whether the higher susceptibility of cotton rats is related to changes in eosinophil functions in response to MF, we examined whether eosinophils from cotton rats form extracellular traps and affect MF motility in a similar manner to eosinophils from BALB/c mice. Eosinophils were generated from the bone marrow of cotton rats and the purity, as determined by Giemsa staining, was >90%. Importantly, eosinophils from mice and cotton rats had granules in the majority of cells (100% of mouse eosinophils, 88% of cotton rat eosinophils) and a distinct circular or segmented nucleus indicating that the cells used were equal in maturity (S2B-C Fig). A representative picture of the generated eosinophils is shown in Fig 4A. Eosinophils from cotton rats reduced the MF motility within the first 48 hours of co-culture. However, after 72 hours, the movements of the MF were still continuous (mean score 3.23) and higher than of MF cultured in the absence of eosinophils (mean score 2.97; Fig 4B). Importantly, MF co-cultured with eosinophils from cotton rats showed no PI staining, indicating their viability (S4D Fig). To investigate whether eosinophils of naïve cotton rats are capable of undergoing EETosis, we next measured the presence of free extracellular DNA in the culture supernatants 24h after stimulation with different numbers of MF and concentrations of zymosan and PMA as positive controls. As shown in Figs 4C and S7, eosinophils from cotton rats are capable of undergoing EETosis, as stimulation with PMA and zymosan resulted in an increased release of extracellular DNA, which was reduced after DNase treatment. This observation was confirmed by DAPI staining and fluorescence microscopy (S8 Fig). In contrast, stimulation with 2500, 10,000, 20,000 and 40,000 MF did not result in extracellular DNA release after 24 h and 72 h of stimulation (Fig 4D-E). These results indicate that MF do not induce extracellular trap formations by eosinophils of the natural host. We hypothesize that this could present a parasite-host adaptation that explains the higher susceptibility of cotton rats to infections with *L. sigmodontis* or intrinsic differences in cotton rat and mouse eosinophils.

## Discussion

ETosis has gained prominence in the study of the effector functions of eosinophils. This particular form of cell death involves the release of decondensed nuclear or mitochondrial DNA into the surrounding environment, with the primary purpose of trapping pathogens to promote their elimination [1,38–40]. Studies focusing on eosinophil ETosis in the context of helminth infections are limited as most studies have focused on neutrophil extracellular traps, which have been shown to play a role in various helminth species such as *Dirofilaria immitis* [41]*, Haemonchus contortus* [42], *Strongyloides stercoralis* [43], *Nippostrongylus brasiliensis* [44,45] and *L. sigmodontis* [46]. These studies indicate that neutrophils mediate NETosis-dependent trapping of L3s, preventing successful migration to the site of infection. The role of eosinophils in this context, however, remains understudied. Eosinophilia and increased levels of their granule proteins were primarily observed after treatment of onchocerciasis and loiasis patients with microfilaricidal drugs as well as during acute filarial pathology associated with the death of MF, such as dermatitis in hyper-responsive onchocerciasis patients or patients with tropical pulmonary eosinophilia [1–3,8–10,15–17]. However, the role of EETosis and the effect of specific eosinophil granule proteins on MF viability and development of pathology requires further research.

Previously, Ehrens et al. demonstrated that MF from both *L. sigmodontis* and *D. immitis* trigger EETosis and *L. sigmodontis* MF-induced ETosis is dependent on dectin-1-, AIM2-, caspase-1-, ASC-, and GSDMD [31,32]. MF covered in eosinophil DNA traps were immobilized and therefore removed more efficiently *in vivo* [31]. Since eosinophil traps are known to also contain cytotoxic granule proteins, we investigated whether the two major granule proteins of eosinophils, MBP and EPO, support the killing of MF after they are immobilized by DNA traps. Previous studies with 129/SvJ WT mice lacking either MBP or EPO showed that deficiency of these granule proteins leads to a significantly increased *L. sigmodontis* adult worm burden after 28 dpi [30]. However, worms were cleared from the EPO-KO, MBP-KO and WT 129/SvJ mice prior to the onset of microfilaremia, preventing the analysis of the effects of both granule proteins on the MF stage.

Results from the present *in vitro* study demonstrate that EPO and MBP inhibit MF motility in a dose-dependent manner. Using comparable concentrations of EPO and MBP, EPO was significantly more potent in inhibiting MF motility in comparison to MBP. Thus, EPO may be, at comparable concentrations to MBP, more toxic for MF. This is supported by a previous study by Hamann et al., who observed a more efficient *in vitro* killing of *Brugia pahangi* and *B. malayi* MF by purified EPO in comparison to purified MBP or ECP [25]. While these findings indicate a protective effect of eosinophil granules against MF per se, they do not consider that MBP is more abundant in eosinophils and comprises almost half of the mass of secondary granules in eosinophils [47] and lack the interaction with other eosinophil effector functions. To address this limitation, the present study used co-cultures of MF and bone marrow-derived eosinophils of EPO KO, MBP KO and WT mice. Eosinophils from both KO mouse strains had the capacity to undergo EETosis and form DNA traps, but were less efficient at immobilizing MF compared to WT eosinophils. In addition, potential killing of MF, as assessed by PI staining after 72 hours of co-culture and quantification of the frequency of completely immobilized MF, was more prominent in co-cultures with WT eosinophils than KO eosinophils. Of note, MF motility inhibition and potential killing of MF was comparable for EPO KO and MBP KO eosinophils, indicating a comparable impact of both granule proteins upon release by eosinophils. Given that the lack of MBP or EPO did not completely prevent the motility inhibition and potential killing of MF, it can be hypothesized that redundancy of both granule proteins and potentially other eosinophil granules, such as ECP and EDN, exists and determines the microfilaricidal effect. Future studies should investigate differences in the induction of EETosis in response to MF of different filarial species, e.g., *L. loa*, *Mansonella perstans* and *O. volvulus*, as well as patients with or without filarial pathology, to determine whether EETosis is associated with the development of filarial pathology.

Our experiments on the stimulation of eosinophils from the bone marrow of cotton rats indicate that differences and potential host-specific adaptations concerning the induction of EETosis exist. We demonstrate that cotton rat eosinophils are generally capable of undergoing EETosis. Although, based on the normalized DNA, cotton rat eosinophils release less DNA in response to zymosan and PMA compared to mouse eosinophils. Importantly, MF-induced EETosis was not observed in cotton rat eosinophils in contrast to our findings with mouse eosinophils. This may indicate either intrinsic differences in eosinophil responses from cotton rats and mice or a distinct adaptation of the parasite to its natural host. Thus, *L. sigmodontis* MF may counteract the effects of eosinophils in its natural host, which may increase the susceptibility of cotton rats, resulting in a significantly higher MF load and a longer patency period than in mice. One such potential mechanism was identified by Bouchery et al. who demonstrated that L3 larvae of the murine nematode *N. brasiliensis* secrete DNase to degrade traps formed by neutrophils in mice [45]. Our observation that murine eosinophils are more reactive toward *L. sigmodontis* than comparable eosinophils from the natural host is in line to observations with *B. malayi* and *O. lienalis* that naturally occur in humans and cattle, respectively, and elicit significantly stronger immune responses in the rodent model [48,49]. These differences highlight the potential adaptation of the natural host to chronic filarial infections and the wide variety of immune evasion strategies that were discovered for filariae and helminths in general [50].

These findings demonstrate the need for further research on the natural host to gain a better understanding on the host-parasite relationship, particularly with regard to human disease. It is conceivable that studies on surrogate models of the natural host, such as *O. ochengi* [51], could clarify the question of why some human-pathogenic filarial species lead to the development of filarial pathology, e.g., in onchocerciasis and lymphatic filariasis, whereas others lead mainly to asymptomatic infections, e.g., in *Mansonella perstans* infections [52].

## Supporting information

S1 Fig Purity of murine eosinophils.
Representative flow cytometry-plot displaying the purity of murine bone marrow-derived eosinophils. Eosinophils were identified as SiglecF+ and CD11b +. Additional cell populations in the bone marrow-derived eosinophil culture are shown in the table below.
(TIF)

S2 Fig Purity of cotton rat eosinophils.
Cell composition of the cotton rat eosinophil culture as determined by Diff Quick staining (A). Cotton rat and mouse eosinophils were stained with wheat-germ-agglutinin (WGA) to identify the granules and DAPI to depict the morphology of the nucleus (B). White stars indicate cotton rat eosinophils that do not display granules (10 cells out of 117 total, 11.7%). (C) Zoomed in image of a comparison between cells that show granules and those that do not (white stars).
(TIF)

S3 Fig Percentage of completely immobile microfilariae.
Percentage of completely immobile (score 0) microfilariae (MF) of co-cultures with 10000 MF (A). Pooled data from 5 independent experiments with a total of n = 300 MF (3 wells per experiment with 20 MF each). Motility was documented per condition, not per well. Data were analyzed using two-way ANOVA followed by Bonferroni multiple comparisons test. * p<0.05, ** p<0.01, ns = non-significant.
(TIF)

S4 Fig Mice deficient for EPO or MBP, and the natural host are less capable of killing microfilariae.
Propidium iodide (PI) staining of bone marrow-derived eosinophils co-cultured with microfilariae (MF) for 72h. Microscopic pictures (magnification 100x) of SvJ wildtype **(A)**, MBP KO **(B)**, EPO KO **(C)** and cotton rat eosinophils **(D)** after 72 hours of incubation with MF (Transmitted Light left, PI middle, overlay right). The pictures are representative for five experiments.
(TIF)

S5 Fig MBP KO and EPO KO mouse eosinophils are less effective in killing microfilariae than WT eosinophils.
Percentage of propidium iodide positive stained microfilariae (MF) after 24h (A) and 72h (B) of co-culture with bone marrow-derived eosinophils. Data is shown as median with interquartile range. The data were pooled from 3 (A, n=9 images) and 2 (B, n=6 images) independent experiments. Data were analyzed using Kruskal-Wallis test followed by Dunn´s multiple comparisons test for selected groups for non-parametric data (A-B). *p<0.05, ns = non-significant.
(TIF)

S6 Fig Comparison of microfilariae counts over time between BALB/c mice and cotton rats.
Blood microfilariae counts in BALB/c mice from day 58 to day 113 post infection **(A)**. Blood microfilariae counts in cotton rats from 3 months post infection to 19 months post infection **(B)**. Data is shown as mean and SEM (A-B). Pooled data n = 5-16 mice (A) and n = 1-76 cotton rats (B) per data point.
(TIF)

S7 Fig Eosinophils of the natural host are able to perform ETosis in response to PMA and zymosan.
Free DNA in supernatant of bone marrow-derived cotton rat eosinophils stimulated for 24h or 72h with increasing concentrations of PMA or zymosan. Single experiment with n= 80 microfilariae/ 4 wells. Data is shown as median with interquartile range.
(TIF)

S8 Fig Eosinophils of the natural host can perform EETosis, but not in response to microfilariae.
DAPI staining of bone marrow-derived eosinophils co-cultured with PMA (A), zymosan (B) or 2500 microfilariae (MF) (C) for 24h. The pictures are representative of two experiments. 200x magnification.
(TIF)

S1 Table Original data sets shown in Fig 1, Fig 2B-F , Fig 3A-D , Fig 4B-E, S2A Fig, S3 Fig, S5 Fig A-B, S6A-B Fig, S7A-D Fig.
(XLSX)
