# Supplementary figures and images for: Major basic protein and eosinophil peroxidase support microfilariae motility inhibition by eosinophil ETosis
Source: PLoS Negl Trop Dis. 2025 Mar 3;19(3):e0012889. doi: 10.1371/journal.pntd.0012889 (PMC11902130; doi:10.1371/journal.pntd.0012889)

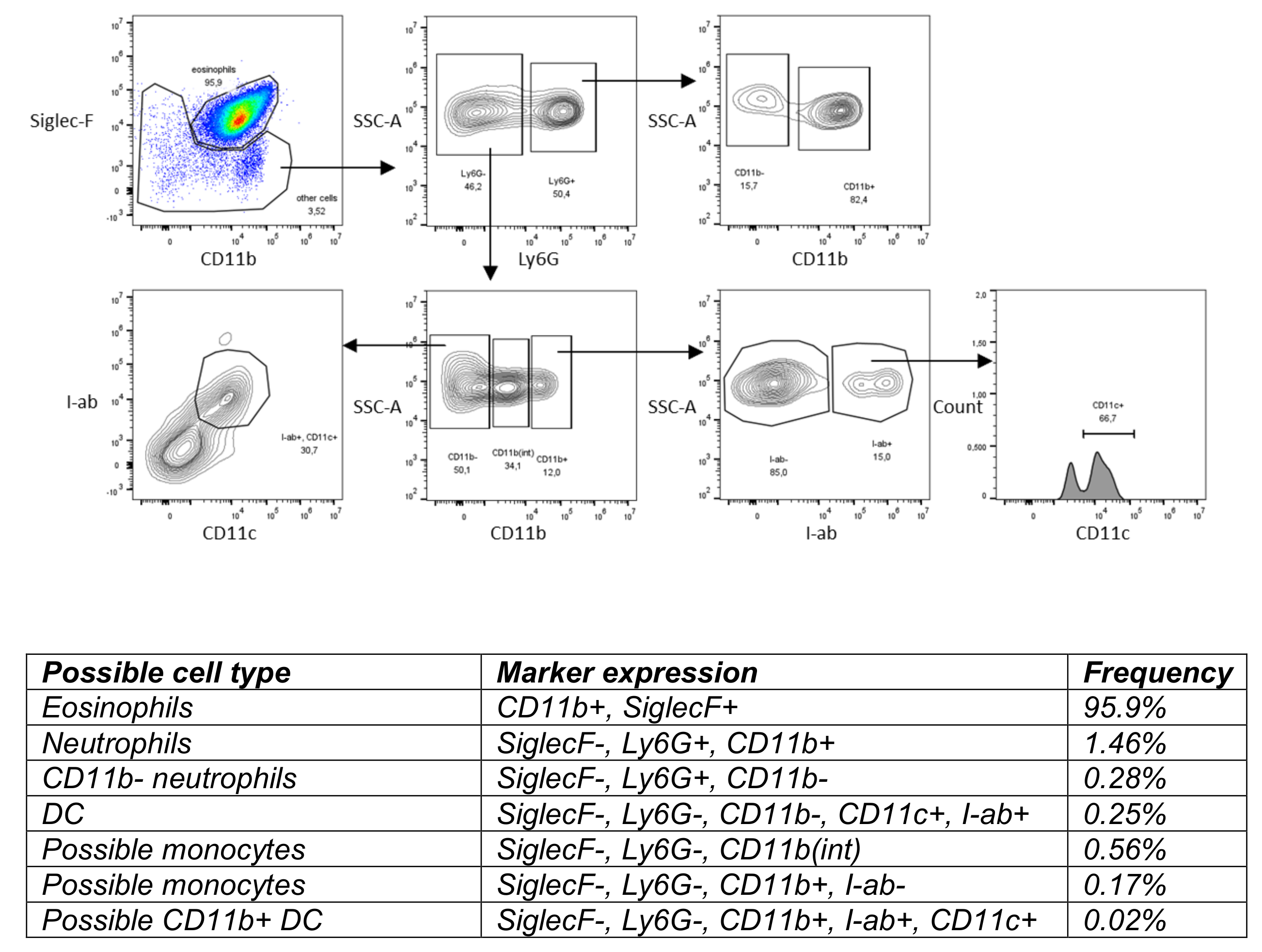

Supplement: S1 Fig — Representative flow cytometry-plot displaying the purity of murine bone marrow-derived eosinophils. Eosinophils were identified as SiglecF+ and CD11b +. Additional cell populations in the bone marrow-derived eosinophil culture are shown in the table below. (TIF) [file pntd.0012889.s001.tif]

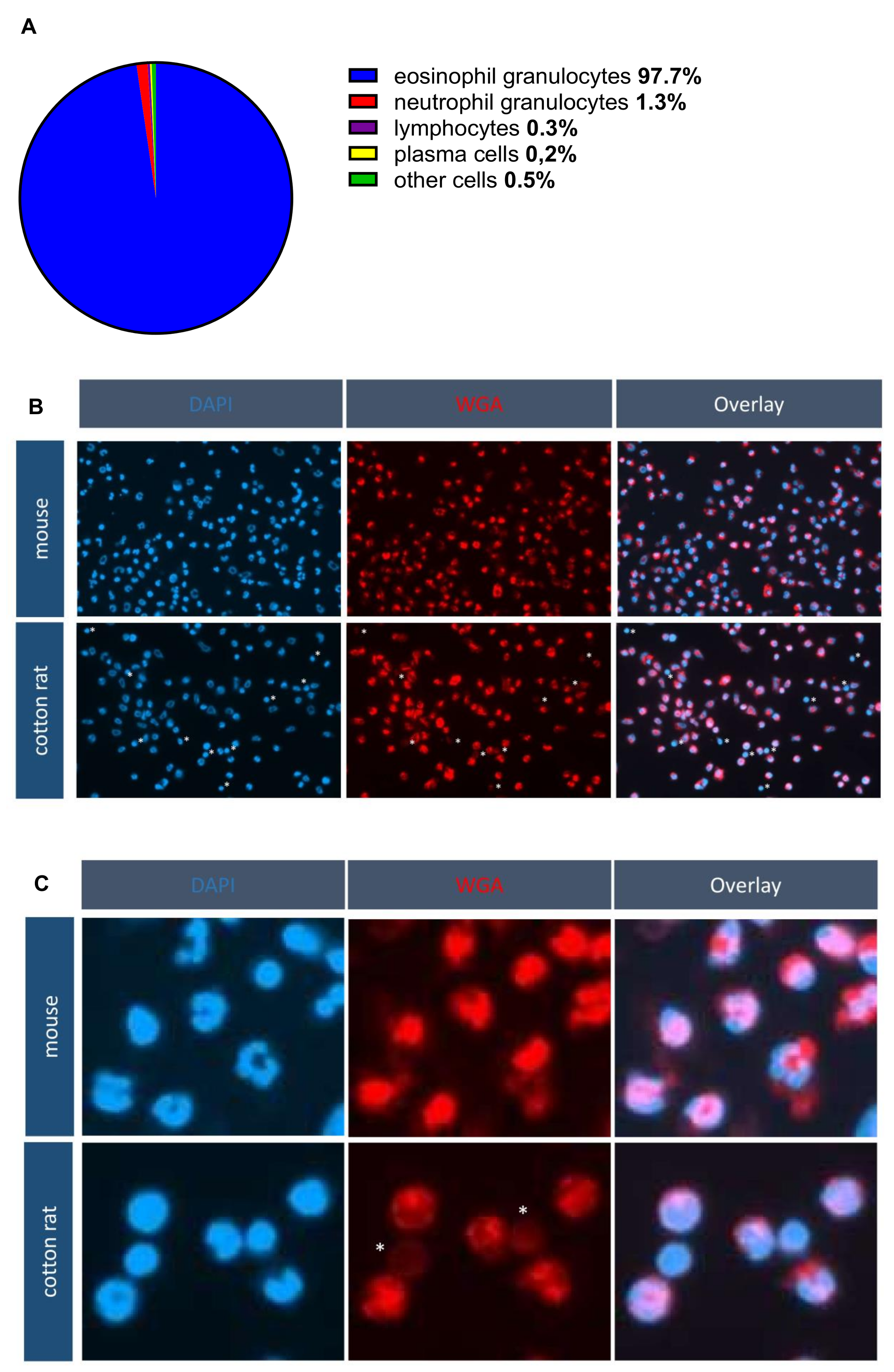

Supplement: S2 Fig — Cell composition of the cotton rat eosinophil culture as determined by Diff Quick staining (A). Cotton rat and mouse eosinophils were stained with wheat-germ-agglutinin (WGA) to identify the granules and DAPI to depict the morphology of the nucleus (B). White stars indicate cotton rat eosinophils that do not display granules (10 cells out of 117 total, 11.7%). (C) Zoomed in image of a comparison between cells that show granules and those that do not (white stars). (TIF) [file pntd.0012889.s002.tif]

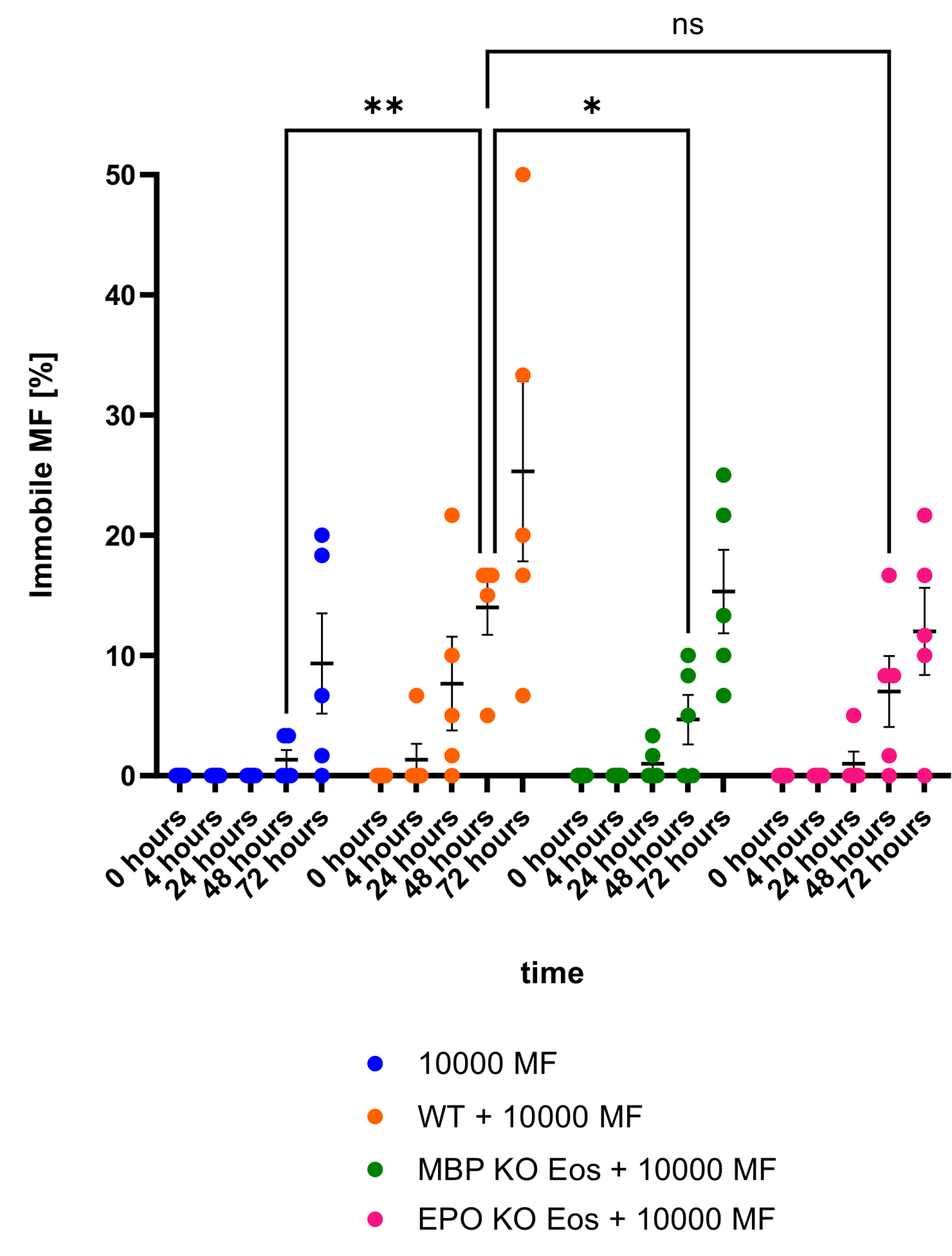

Supplement: S3 Fig — Percentage of completely immobile (score 0) microfilariae (MF) of co-cultures with 10000 MF (A). Pooled data from 5 independent experiments with a total of n = 300 MF (3 wells per experiment with 20 MF each). Motility was documented per condition, not per well. Data were analyzed using two-way ANOVA followed by Bonferroni multiple comparisons test. * p<0.05, ** p<0.01, ns = non-significant. (TIF) [file pntd.0012889.s003.tif]

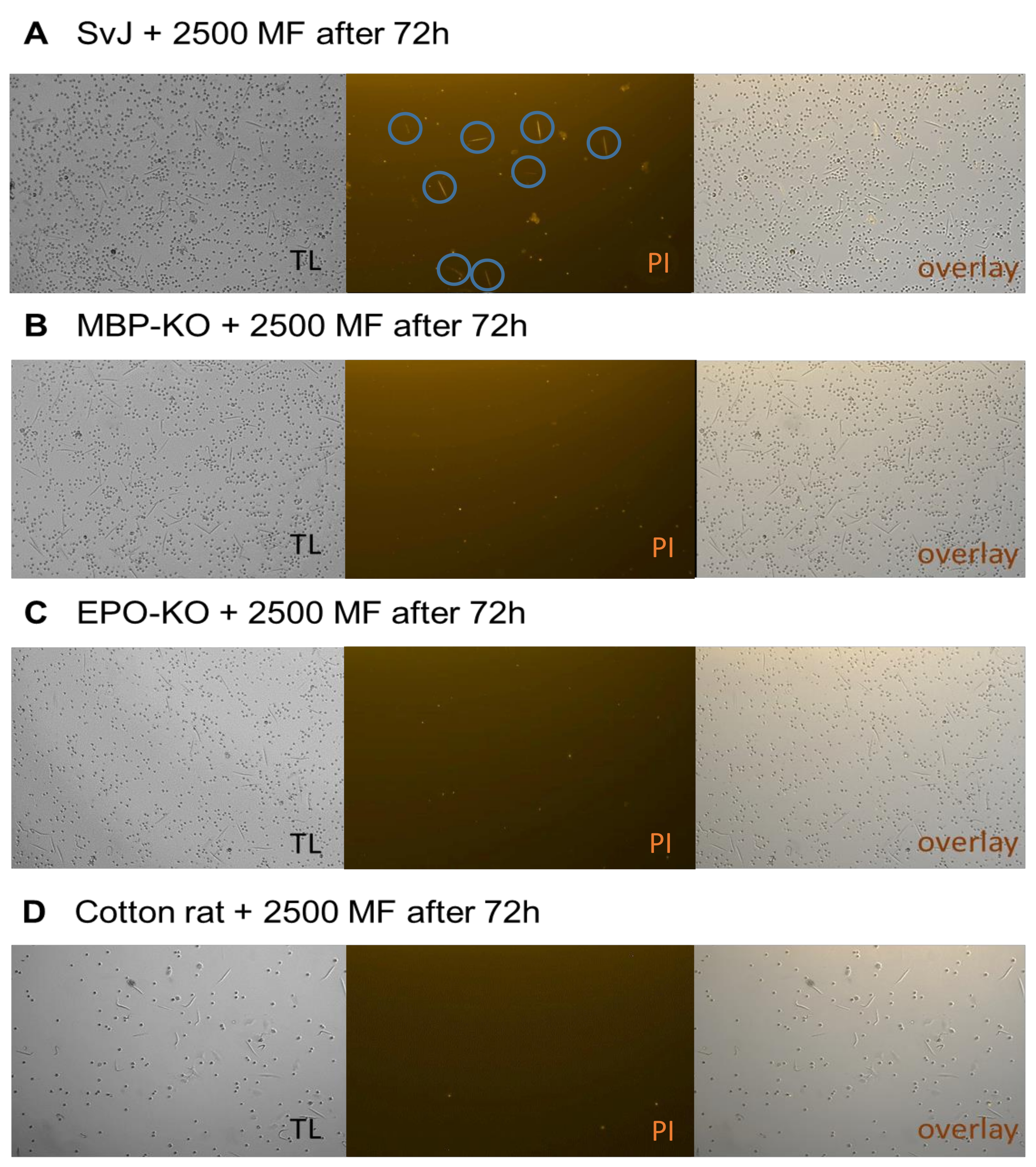

Supplement: S4 Fig — Propidium iodide (PI) staining of bone marrow-derived eosinophils co-cultured with microfilariae (MF) for 72h. Microscopic pictures (magnification 100x) of SvJ wildtype (A), MBP KO (B), EPO KO (C) and cotton rat eosinophils (D) after 72 hours of incubation with MF (Transmitted Light left, PI middle, overlay right). The pictures are representative for five experiments. (TIF) [file pntd.0012889.s004.tif]

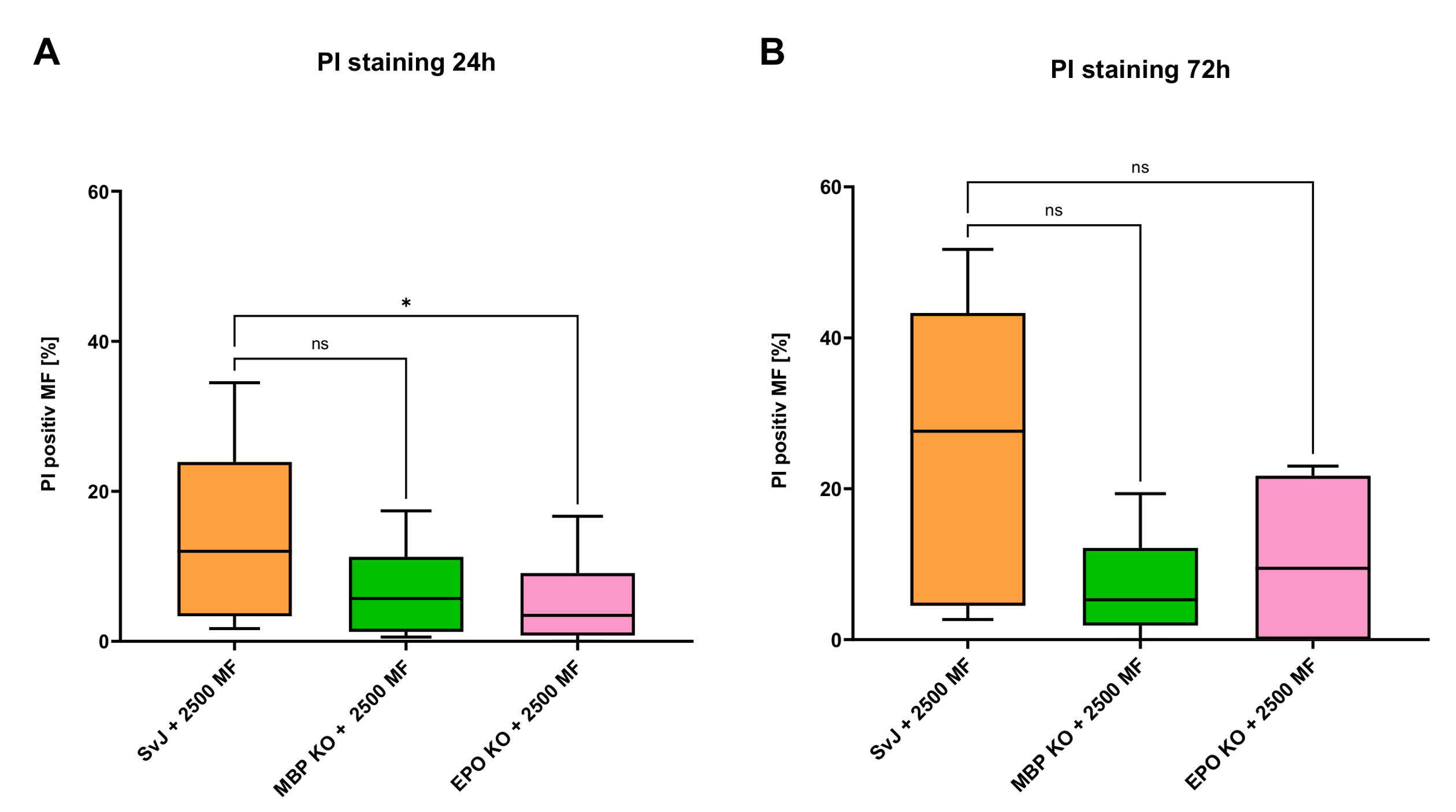

Supplement: S5 Fig — Percentage of propidium iodide positive stained microfilariae (MF) after 24h (A) and 72h (B) of co-culture with bone marrow-derived eosinophils. Data is shown as median with interquartile range. The data were pooled from 3 (A, n=9 images) and 2 (B, n=6 images) independent experiments. Data were analyzed using Kruskal-Wallis test followed by Dunn´s multiple comparisons test for selected groups for non-parametric data (A-B). *p<0.05, ns = non-significant. (TIF) [file pntd.0012889.s005.tif]

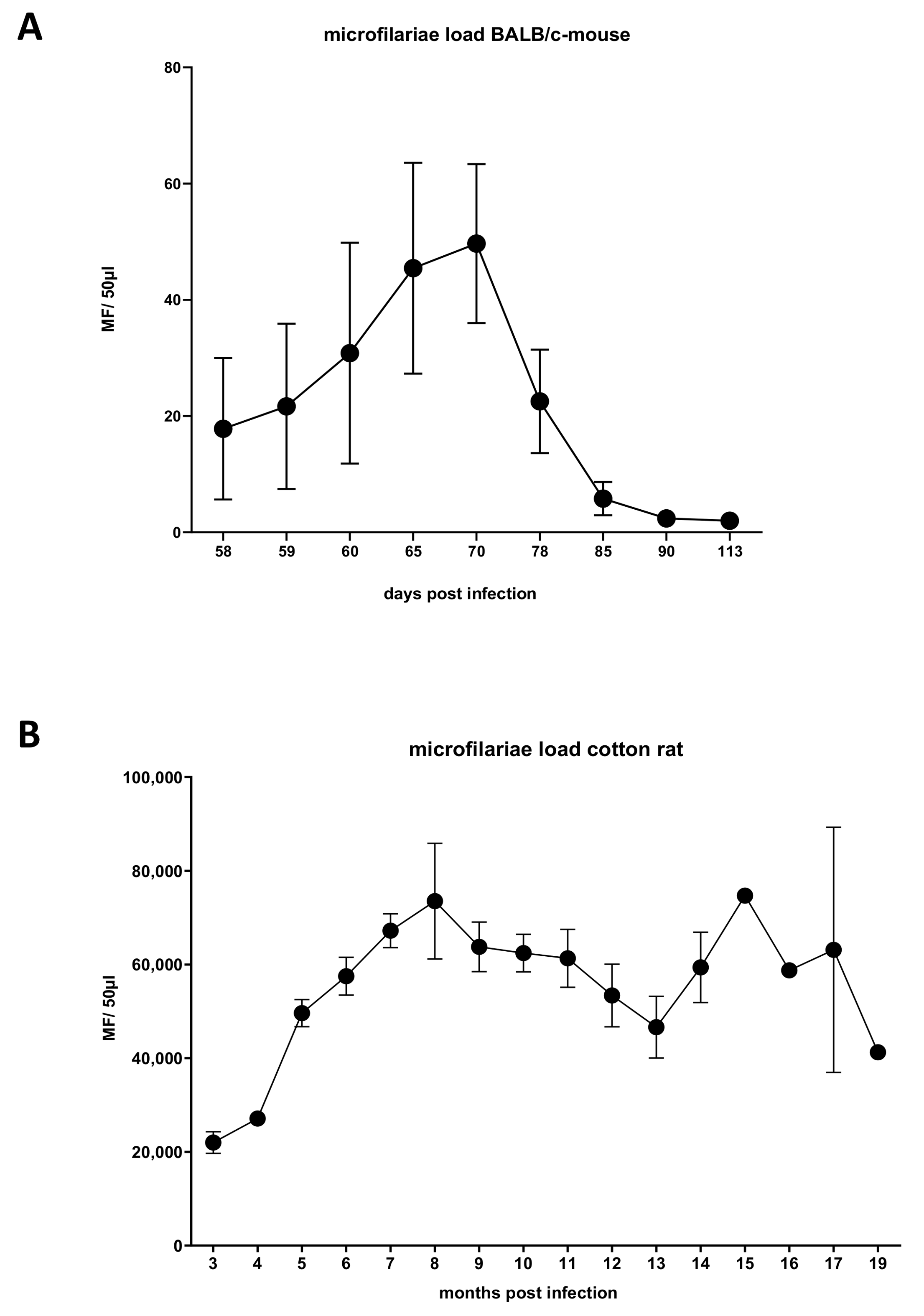

Supplement: S6 Fig — Blood microfilariae counts in BALB/c mice from day 58 to day 113 post infection (A). Blood microfilariae counts in cotton rats from 3 months post infection to 19 months post infection (B). Data is shown as mean and SEM (A-B). Pooled data n = 5-16 mice (A) and n = 1-76 cotton rats (B) per data point. (TIF) [file pntd.0012889.s006.tif]

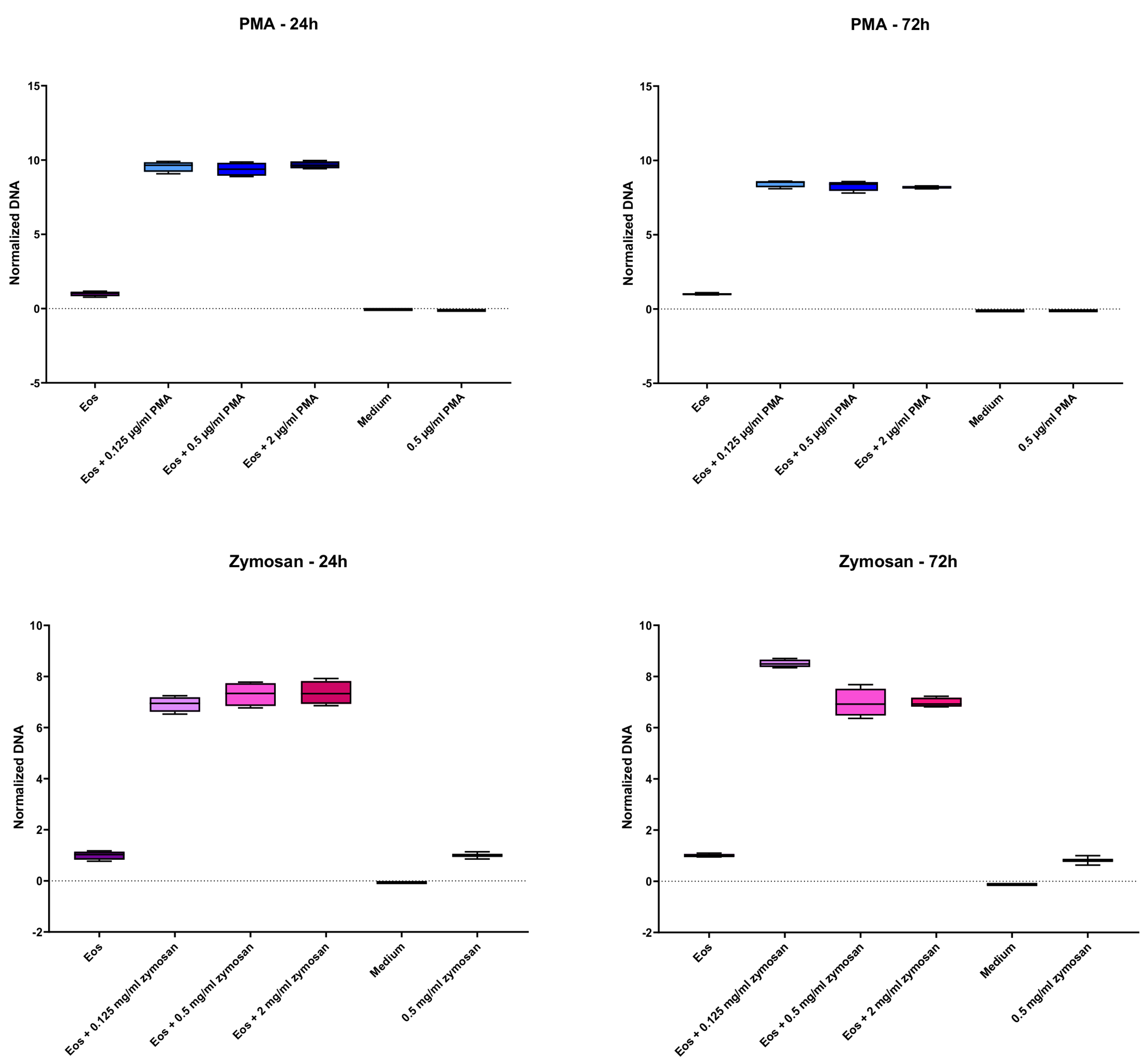

Supplement: S7 Fig — Free DNA in supernatant of bone marrow-derived cotton rat eosinophils stimulated for 24h or 72h with increasing concentrations of PMA or zymosan. Single experiment with n= 80 microfilariae/ 4 wells. Data is shown as median with interquartile range. (TIF) [file pntd.0012889.s007.tif]

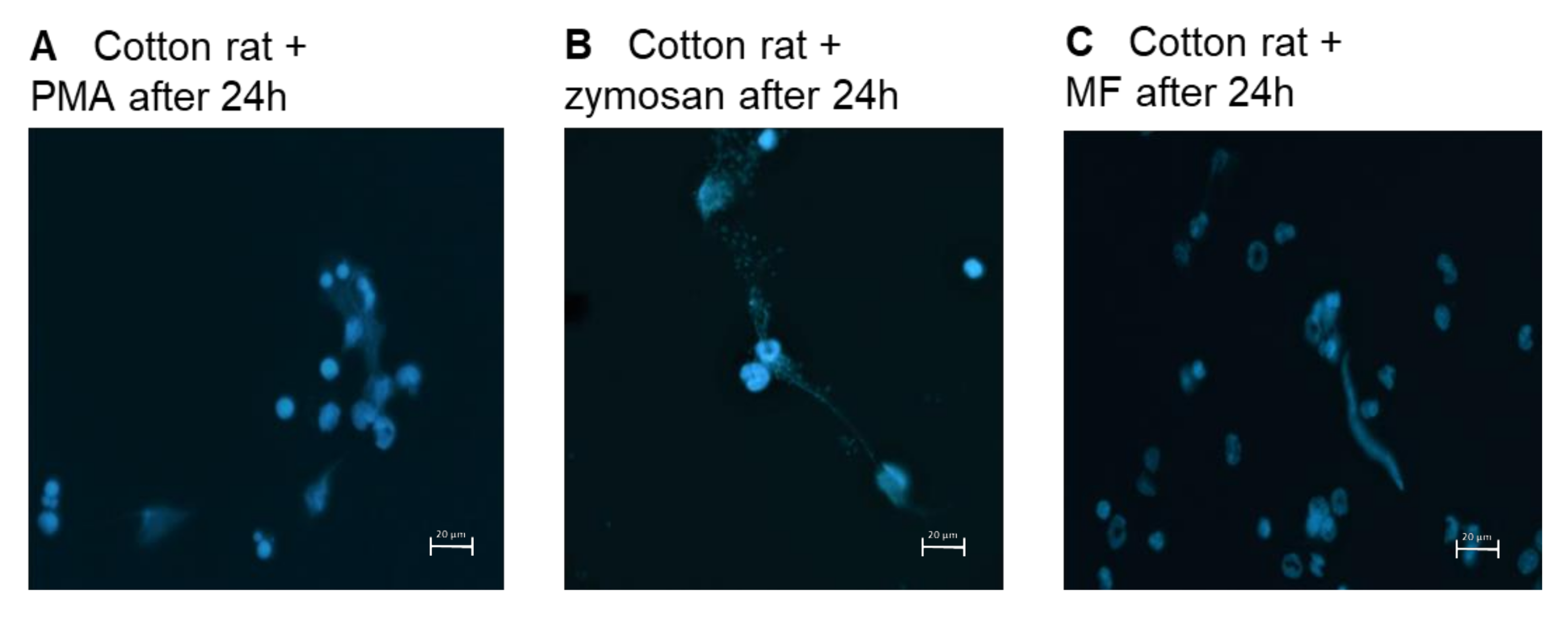

Supplement: S8 Fig — DAPI staining of bone marrow-derived eosinophils co-cultured with PMA (A), zymosan (B) or 2500 microfilariae (MF) (C) for 24h. The pictures are representative of two experiments. 200x magnification. (TIF) [file pntd.0012889.s008.tif]
